# Melioidosis in a Tropical City State, Singapore

**DOI:** 10.3201/eid1510.090246

**Published:** 2009-10

**Authors:** Tong Jen Lo, Li Wei Ang, Lyn James, Kee Tai Goh

**Affiliations:** Ministry of Health, Singapore

**Keywords:** Melioidosis, Singapore, bacteria, dispatch

## Abstract

The incidence of melioidosis in Singapore decreased during 1998–2007, with the exception of the first quarter of 2004. After heavy rainfalls, an increase in pneumonic cases with a high case-fatality rate was detected. We show that melioidosis has the potential to reemerge following adverse climate events.

Melioidosis is a tropical infectious disease caused by a gram-negative bacillus, *Burkholderia pseudomallei*. It is endemic to southeast Asia and northern Australia, and cases are increasingly being reported in countries elsewhere in Asia, the Pacific, the Americas, the Caribbean, Africa, and the Middle East, and in travelers returning from tropical countries (*1*).

*B. pseudomallei* is a saprophytic bacterium that can be found in soil and water samples in melioidosis-endemic countries. Transmission is generally by direct inoculation from exposure to soil or water, or through inhalation of aerosolized particles. The disease often affects persons with underlying conditions such as diabetes mellitus (*1*,*2*). Clinical manifestations are protean and may range from chronic abscesses to fulminant pneumonia and septicemia with high death rates (*1*).

Singapore is a tropical island city state in southeast Asia. More than 80% of the population lives in high-rise public housing estates. Although the first case of melioidosis in Singapore was reported in 1920 (*3*), little is known about the incidence of the disease before it was made notifiable in 1989 when 3 apparently healthy young men died from melioidosis (*4*). We studied the epidemiology and clinical features of melioidosis in Singapore over a 10-year period (1998–2007). Our study assessed the trends in the epidemiology of the disease, clinical features, case-fatality rates, and risk factors associated with death.

## The Study

We analyzed the epidemiologic data of all cases of melioidosis reported by registered medical practitioners and laboratories to the Singapore Ministry of Health during 1998–2007. Clinical and laboratory criteria for notification were based on guidelines disseminated by the Ministry (*5*). Upon notification, trained public health officers carried out epidemiologic investigations by using a standardized form. Investigations included interviews with the patient or family members and a review of hospital records and laboratory results. Data obtained included age, gender, date of onset of the illness, travel history, possible occupational or recreational exposure to contaminated soil or water, concurrent medical conditions, laboratory and microbiologic results, and clinical outcome.

Clinical specimens from patients with melioidosis were sent to clinical laboratories in the admitting hospitals for culture of *B. pseudomallei* (*6*) and, in some cases, for serology. Isolates were tested for antimicrobial sensitivity (*7*). A definitive case of melioidosis was defined as a clinically compatible case in which *B. pseudomallei* was isolated from a clinical specimen. If the diagnosis was based on an indirect hemagglutination test that showed a titer >16 (*8*), the case was considered presumptive. Both definitive and presumptive cases were included in the analysis.

The estimated midyear population used for the calculation of the incidence rate was obtained from the Singapore Department of Statistics. Data on rainfall was obtained from the Meteorological Services Division of the National Environment Agency. Foreigners seeking medical treatment for melioidosis were excluded from the data analysis. Statistical analyses were performed by using SPSS Software version 15.0 (SPSS Inc., Chicago, IL, USA). Biivariate analysis was performed by using χ^2^ test for categorical data. A p value ≤0.05 was considered statistically significant.

**Figure 1:**
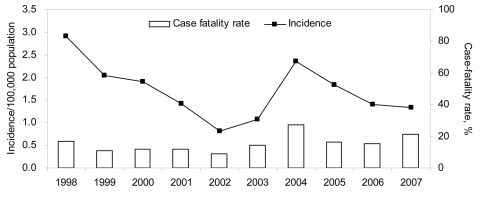
Incidence (per 100,000 population) and case-fatality rate (%) of melioidosis cases, Singapore, 1998–2007.

**Figure 2:**
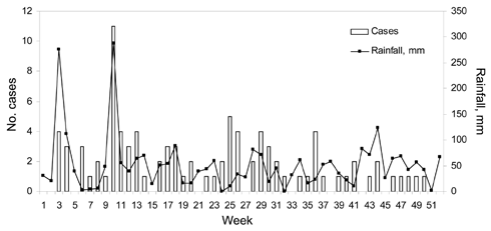
Weekly melioidosis cases by onset date and rainfall totals, Singapore, January 4, 2004–January 1, 2005

A total of 693 cases of melioidosis were reported during 1998–2007; of these, 83% were diagnosed by culture and 17% by serologic analysis. We observed a decreasing trend in the annual incidence rate, with the exception of an increase in 2004 (Figure 1). Patients ranged in age from 1 month to 97 years. The highest age-specific incidence rate of melioidosis was for adults >45 years of age. The annual incidence rate for male patients was 2.8–7.2× that for female. An increase in the number of cases in March and April 2004 was preceded by heavy rainfall (Figure 2), strong winds, and flash floods. A total of 23 cases of melioidosis were reported with onset of illness during the 5-week period between March 7 and April 10 (epidemiologic weeks 10–14). The proportion of cases with the pneumonic form of melioidosis during this period was 82.6%, compared to 47.8% for the remainder of 2004. The case-fatality rate was 52.6% and 36.4%, respectively (*9*). Only 4 of the 23 case-patients (17.3%) reported occupations that had exposure to soil, e.g., construction workers or gardeners.

A total of 112 deaths were reported during the 10-year period; overall case-fatality rate was 16.2% (range 8.8%–27.1%). Of the reported cases, 75.5% had co-existing diseases, with diabetes (47.9%), hypertension (26.4%), renal impairment (13.3%), and ischemic heart disease (12.0%) being the most common. Patients with co-illnesses had a significantly higher case-fatality rate (19.3%) compared to those without (6.5%) (p<0.0005). Approximately half (50.4%) of the melioidosis cases were associated with bacteremia. Patients with bacteremic melioidosis had a significantly higher case-fatality rate (25.8%) than those without bacteremia (5.5%) (p<0.0005). Clinical isolates of *B. pseudomallei* demonstrated antimicrobial sensitivity to imipenem (100.0%), ceftazidime (99.1%), doxycycline (99.0%), amoxicillin/clavulanate (94.2%), and chloramphenicol (96.1%).

## Conclusions

Our study showed that male gender, old age, and diabetes mellitus were risk factors for melioidosis. The presence of bacteremia and co-illnesses were risk factors for death in patients with melioidosis, consistent with findings in other endemic countries. The overall case-fatality rate in this study was much lower compared to cases during 1989–1996 (39.5%) (*10*). This may be due to greater awareness among medical practitioners, earlier recognition of the disease, better intensive care, and an appropriate antimicrobial drug regimen.

Unlike patients in Australia or Thailand, most of the case-patients in our study could not recall any occupational or recreational exposure to wet soil. The only reported episodes of percutaneous inoculation in Singapore were a few young adults with localized cutaneous infections and abscesses caused by occupational exposure to soil (*4*). *B. pseudomallei* was isolated from only 1.8% of soil samples and from none of the water samples in Singapore collected during epidemiologic investigations of reported cases (*11*). Tan et al. have suggested that in an urban setting excessive soil excavations could contribute to aerosolization of the bacterium (*12*).

Rapid molecular typing of the bacteria during the outbreak in 2004 showed that the isolates were genetically heterogeneous, thus excluding the possibility of a common source (*13*). Moreover, the cases were distributed in different parts of the island without any particular geographic predilection. An epidemiologic investigation of cases in the first half of 2004 demonstrated a relationship between incidence of melioidosis and cumulative rainfall 7 days before onset of illness (*14*). This finding is consistent with other studies that demonstrated an association between incidence of melioidosis and intensity of rainfall. Researchers have posited that heavy rainfall causes movement of the bacteria to the surface with the rising water table (*15*). Severe climatic events may cause aerosolization of the bacteria and increase the risk for inhalation. Infection following inhalation of *B. pseudomallei* may result in more fulminant disease and a higher case-fatality rate (*15*).

Melioidosis is emerging as a serious public health problem in many countries. Although the incidence and case-fatality rate of melioidosis in Singapore has decreased, it has the potential to resurface with adverse climate events such as heavy rainfall and flash floods.
